# Supplementary material for: Isolation and Characterization of a Novel Siphoviridae Phage, vB_AbaS_TCUP2199, Infecting Multidrug-Resistant Acinetobacter baumannii
Source: Viruses. 2022 Jun 7;14(6):1240. doi: 10.3390/v14061240 (PMC9228384; doi:10.3390/v14061240)
Supplement: Supplementary file 1 [file viruses-14-01240-s001.zip › Supplementary Table S1.pdf]

Supplementary Table S1. Host range analysis of TCUP2199

Strain	Source	Infection
TV64	TVGH	++
TV101	TVGH	++
TV312	TVGH	++
TV313	TVGH	++
TV318	TVGH	++
TV341	TVGH	++
TV347	TVGH	+
TV354	TVGH	++
TV379	TVGH	++
TV391	TVGH	++
TV408	TVGH	++
TV417	TVGH	++
TV419	TVGH	+
TV424	TVGH	++
TV429	TVGH	++
TV438	TVGH	++
TV441	TVGH	++
TV454	TVGH	++
TV456	TVGH	++
TV479	TVGH	++
TV481	TVGH	-
TV497	TVGH	++
TV514	TVGH	-
TV530	TVGH	-
TV618	TVGH	+
TV664	TVGH	++
TV695	TVGH	++
TV708	TVGH	++
TV757	TVGH	++
TV868	TVGH	-
TV906	TVGH	++
TV937	TVGH	++
TV948	TVGH	++
TV967	TVGH	++
TV999	TVGH	++
TV1003	TVGH	++
TV1019	TVGH	++
TV1033	TVGH	++
TV1052	TVGH	++
TV1089	TVGH	++
TV1124	TVGH	++
TV1131	TVGH	++
TV1288	TVGH	++
TV1532	TVGH	++
TV1544	TVGH	++
TV1729	TVGH	++
TV331	TVGH	++
TV338	TVGH	+
TV346	TVGH	+
TV350	TVGH	++
TV352	TVGH	++
TV363	TVGH	++
TV366	TVGH	++
TV385	TVGH	+
TV406	TVGH	++
TV433	TVGH	+
TV445	TVGH	+
TV452	TVGH	+
TV458	TVGH	++
TV461	TVGH	+
TV467	TVGH	+
TV485	TVGH	++
TV489	TVGH	++
TV555	TVGH	++
TV560	TVGH	++
TV563	TVGH	++
TV574	TVGH	-
TV594	TVGH	++
TV600	TVGH	+
TV612	TVGH	+
TV616	TVGH	++
TV617	TVGH	+
TV619	TVGH	+
TV644	TVGH	+
TV663	TVGH	++
TV690	TVGH	+
TV734	TVGH	+
TV735	TVGH	++
TV746	TVGH	++
TV755	TVGH	++
TV807	TVGH	++
TV818	TVGH	++
TV862	TVGH	++
TV894	TVGH	++
TV1702	TVGH	++
TV1731	TVGH	-
TV1719	TVGH	++
TV1721	TVGH	++
TV1733	TVGH	++
TV1734	TVGH	++
TV1735	TVGH	++
TV1740	TVGH	++
TV1742	TVGH	++
TV1861	TVGH	++
TV2171	TVGH	++
TV2199	TVGH	++
TV2202	TVGH	++
TV2203	TVGH	++
TV2209	TVGH	++
TV2210	TVGH	++

++: clear spot; +: turbid spot; -: no spot (not infected); TVGH: Taipei Veterans General Hospital; HBTZH: Hualien Buddhist Tzu Chi Hospital; ATCC: American Type Culture Collection

Strain	Source	Infection	Strain	Source	Infection	Strain	Source	Infection	Strain	Source	Infection
TV2211	TVGH	++	TV2453	TVGH	++	TV2771	TVGH	++	TV974	TVGH	++
TV2216	TVGH	++	TV2454	TVGH	++	TV2792	TVGH	++	TV991	TVGH	+
TV2222	TVGH	++	TV2455	TVGH	++	TV2804	TVGH	++	TV997	TVGH	-
TV2229	TVGH	++	TV2456	TVGH	++	TV2816	TVGH	++	TV1009	TVGH	-
TV2252	TVGH	++	TV2465	TVGH	++	TV129	TVGH	++	TV1015	TVGH	-
TV2262	TVGH	++	TV1750	TVGH	++	TV130	TVGH	++	TV1016	TVGH	+
TV2265	TVGH	-	TV1855	TVGH	++	TV255	TVGH	++	TV1021	TVGH	-
TV2280	TVGH	-	TV1860	TVGH	++	TV311	TVGH	++	TV1077	TVGH	-
TV2282	TVGH	++	TV1862	TVGH	++	TV315	TVGH	++	TV1027	TVGH	+
TV2289	TVGH	++	TV2177	TVGH	++	TV169	TVGH	++	TV1036	TVGH	+
TV2314	TVGH	++	TV2201	TVGH	++	TV173	TVGH	++	TV1066	TVGH	++
TV2318	TVGH	++	TV2283	TVGH	++	TV317	TVGH	++	TV1080	TVGH	++
TV2326	TVGH	++	TV2410	TVGH	++	TV319	TVGH	++	TV1082	TVGH	++
TV2334	TVGH	++	TV2427	TVGH	++	TV323	TVGH	++	TV1093	TVGH	-
TV2345	TVGH	-	TV2439	TVGH	+	TV908	TVGH	**++**	TV1096	TVGH	+
TV2362	TVGH	++	TV2469	TVGH	-	TV902	TVGH	**++**	TV1573	TVGH	+
TV2367	TVGH	++	TV2588	TVGH	++	TV913	TVGH	**++**	TV1130	TVGH	++
TV2374	TVGH	++	TV2606	TVGH	-	TV916	TVGH	**++**	TV1306	TVGH	++
TV2391	TVGH	++	TV2654	TVGH	++	TV921	TVGH	**++**	TV1316	TVGH	++
TV2401	TVGH	++	TV2700	TVGH	++	TV930	TVGH	**++**	TV1536	TVGH	++
TV2405	TVGH	++	TV2713	TVGH	+	TV940	TVGH	**++**	TV1537	TVGH	++
TV2409	TVGH	++	TV2721	TVGH	+	TV952	TVGH	**++**	TV1539	TVGH	++
TV2422	TVGH	++	TV2724	TVGH	++	TV960	TVGH	**++**	TV1551	TVGH	+
TV2424	TVGH	++	TV2731	TVGH	+	TV978	TVGH	**-**	8 -- 2	HBTZH	+
TV2441	TVGH	++	TV2768	TVGH	-	TV962	TVGH	++	9 -- 3	HBTZH	++

++: clear spot; +: turbid spot; -: no spot (not infected); TVGH: Taipei Veterans General Hospital; HBTZH: Hualien Buddhist Tzu Chi Hospital; ATCC: American Type Culture Collection

Strain	Source	Infection
M3237	HBTZH	++
M68316	HBTZH	+
M6777	HBTZH	+
M68630	HBTZH	++
ATCC17978	ATCC	++
ATCC19606	ATCC	+

++: *clear spot;* +: *turbid spot;* -: *no spot (not infected); TVGH: Taipei Veterans General Hospital; HBTZH: Hualien Buddhist Tzu Chi Hospital; ATCC: American Type Culture Collection*
