# Supplementary material for: Isolation and Characterization of a Novel Siphoviridae Phage, vB_AbaS_TCUP2199, Infecting Multidrug-Resistant Acinetobacter baumannii
Source: Viruses. 2022 Jun 7;14(6):1240. doi: 10.3390/v14061240 (PMC9228384; doi:10.3390/v14061240)
Supplement: Supplementary file 1 [file viruses-14-01240-s001.zip › Supplementary Table S2.pdf]

Supplementary Table S2. General features of the putative ORFs from vB_AbaS_TCUP2199 with the best matches in the database

ORFs	Start	End	Amino acid	Protein size (kDa)	Annotated function	Best homolog	E value	Identity	Query cover	Accession no.
**1**	361	660	99	11.21	No similarity	-	-	-	-	-
**2**	657	1025	122	13.62	No similarity	-	-	-	-	-
**3**	1437	1697	86	10.03	No similarity	-	-	-	-	-
**4**	1770	1940	56	5.56	No similarity	-	-	-	-	-
**5**	2673	2969	98	11.16	No similarity	-	-	-	-	-
**6**	2981	3946	321	37.43	No similarity	-	-	-	-	-
**7**	3939	4151	70	8.09	No similarity	-	-	-	-	-
**8**	4160	4642	160	18.43	No similarity	-	-	-	-	-
**9**	4654	4947	97	10.77	No similarity	-	-	-	-	-
**10**	4959	5378	139	15.55	No similarity	-	-	-	-	-
**11**	5390	5614	74	8.74	No similarity	-	-	-	-	-
**12**	5611	6102	163	18.76	No similarity	-	-	-	-	-
**13**	6099	6509	136	16.25	No similarity	-	-	-	-	-
**14**	6758	7015	85	9.51	DNA binding HTH domain protein	*Vibrio* phage 1.086.O.10N.222.51	9e-05	53.66%	48%	AUR86530 .1
**15**	7026	7523	165	19.31	No similarity	-	-	-	-	-
**6**	7523	7897	124	14.31	No similarity	-	-	-	-	-
**7**	7953	8312	119	13.37	No similarity	-	-	-	-	-
**8**	8312	9910	532	56.59	TPA: MAG TPA: hypothetical protein	*Siphoviridae sp.*	2e-47	45.90%	45%	DAE96214 .1
**19**	9979	10296	105	12.07						
**20**	10318	10830	170	19.46	Hypothetical protein	*Acinetobacter sp.* YH12231	13-11	34.19%	75%	WP_18007 4459.1
**21**	10840	11367	175	20	Hypothetical protein	*Acinetobacter indicus*	2e-10	44.78%	38%	WP_01665 8914.1
**22**	11377	11871	164	19.68	No similarity	-	-	-	-	-
**23**	11868	12617	249	29.99	No similarity	-	-	-	-	-
**24**	12628	13167	179	19.99	No similarity	-	-	-	-	-

**25**	13164	13427	87	10.03	No similarity	-	-	-	-	-
**26**	13430	13858	142	16.05	No similarity	-	-	-	-	-
**27**	13875	14183	102	11.94	No similarity	-	-	-	-	-
**28**	14195	14734	179	20.45	No similarity	-	-	-	-	-
**29**	14780	15340	186	21.2	Hypothetical protein HWB88_gp72	*Klebsiella* phage Seifer	73-18	40%	79%	YP_009841 609.1
**30**	15337	15501	54	5.74	No similarity	-	-	-	-	-
**31**	15502	15939	145	16.75	No similarity	-	-	-	-	-
**32**	15936	16292	118	13.35	No similarity	-	-	-	-	-
**33**	16267	16917	216	24.22	No similarity	-	-	-	-	-
**34**	17022	17444	140	15.15	Hypothetical protein	*Pseudomonas sp.* PDM29	1e-12	42.71%	68%	WP_21854 3825.1
**35**	17498	18355	285	32.55	DUF2303 family protein	*Acinetobacter sp.* YH12025	8e-16	27.48%	77%	WP_18017 5460.1
**36**	18433	18846	137	15.83	No similarity	-	-	-	-	-
**37**	18859	19314	151	16.86	No similarity	-	-	-	-	-
**38**	19344	19547	67	7.34	No similarity	-	-	-	-	-
**39**	19544	19828	94	10.98	No similarity	-	-	-	-	-
**40**	19837	20310	157	18.4	No similarity	-	-	-	-	-
**41**	20328	21905	525	60.19	DEAD-like helicase	*Pseudoalteromonas* phage KB12-38	3e-56	30.30%	91%	ASV43993. 1
**42**	22040	22825	261	27.36	No similarity	-	-	-	-	-
**43**	22841	23056	71	8.25	No similarity	-	-	-	-	-
**44**	23044	23862	272	31.12	DNA polymerase I thermostable	*Achromobacter* phage JWF	6e-16	32.26%	87%	YP_009224 065.1
**45**	23859	24398	179	19.20	No similarity	-	-	-	-	-
**46**	24462	26561	699	80.17	Phage capsid protein	*Acinetobacter seifertii*	3e-147	40.13%	88%	WP_20002 8987.1
**47**	26571	27569	332	37.28	Tail fiber protein	*Xanthomonas* phage XAJ24	3e-18	30.64%	50%	YP_009785 951.1
**48**	27573	28664	363	40.08	DUF285 domain-containing protein	*Acinetobacter sp.* ANC3789	6e-20	37.41%	39%	WP_00475 2440.1
**49**	28682	29554	290	31.93	No similarity	-	-	-	-	-

**50**	29639	30061	140	15.5	No similarity	-	-	-	-	-
**51**	30077	30268	63	7.23	No similarity	-	-	-	-	-
**52**	30427	31140	237	27.08	Endolysin	*Caulobacter* phage Seuss	7e-27	34.93%	95%	YP_009785 554.1
**53**	31156	31305	49	5.44	No similarity	-	-	-	-	-
**54**	32924	33223	99	11.09	No similarity	-	-	-	-	-
**55**	33216	33407	63	6.6	No similarity	-	-	-	-	-
**56**	33820	36399	859	97.68	DNA polymerase I	*Achromobacter* phage JWF	0	41.94%	98%	YP_009224 062.1
**57**	36392	36739	115	14.4	No similarity	-	-	-	-	-
**58**	36690	37475	261	29.88	No similarity	-	-	-	-	-
**59**	37570	37788	72	8.34	Hypothetical phage	Bacteriophage sp.	3e-18	55.56%	81.3%	QHJ78711. 1
**60**	37791	38318	175	19.98	Hypothetical phage	Bacteriophage sp.	6e-43	53.38%	84%	QHJ78713. 1
**61**	38345	39031	228	26.4	Exonuclease	*Achromobacter* phage JWF	3e-10	25.62%	89%	YP_009224 051.1
**62**	39121	39402	93	10.87	Hypothetical protein	*Acinetobacter baumannii*	3e-17	62.71%	63%	WP_10413 3486.1
**63**	39399	39815	138	15.51	No similarity	-	-	-	-	-
**64**	39823	40950	375	42.4	DNA repair exonuclease	*Pseudoalteromonas* phage KB12-38	3e-26	27.70%	93%	ASV44006. 1
**65**	40943	43117	724	82.95	Nucleoside hydrolase	*Pseudoalteromonas* KB12-38	1e-53	25.53%	98%	ASV44007. 1
**66**	43119	43385	88	10.09	Hypothetical protein Av05_00110	*Escherichia* phage Av-05	5e-04	35.82%	73%	YP_009111 184.1
**67**	43382	43579	65	7.33	No similarity	-	-	-	-	-
**68**	43579	43875	98	11.02	Hypothetical protein	*Pseudoalteromonas* phage H101	5e-08	37.66%	76%	YP_009225 608.1
**69**	43881	44459	192	22.16	No similarity	-	-	-	-	-
**70**	44555	45268	237	26.2	Nucleoside triphosphate pyrophosphohydrolase family protein	*Pontibacterium sp.* N1Y112	7e-07	31.85%	56%	WP_19395 4187.1

**71**	45268	46215	315	36.97	Thymidylate synthase	*Caulobacter* phage Seuss	7e-48	38.67%	88%	YP_009785564.1
**72**	46268	47342	351	38.84	Hypothetical protein	*Escherichia* phage vB_EcoM_ECO1230-10	2e-13	50.57%	24%	YP_009168937.1
**73**	47459	49408	649	73.11	Ribonucleoside-diphosphate reductase	*Acinetobacter* phage VB_ApiP_XC38	0	54.29%	99%	YP_010105021.1
**74**	49398	50459	353	41.68	Ribonucleotide reductase of class Ia (Aerobic) beta subunit	*Acinetobacter* phage VB_ApiP_XC38	4e-115	51.92%	96%	YP_010105019.1
**75**	50496	51497	333	34.87	Phage tail protein	*Acinetobacter tandoii*	5e-24	56.88%	32%	WP_151503848.1
**76**	51549	54938	1129	127.37	Tail fiber protein	*Caulobacter* phage Seuss	1e-36	28.81%	49%	YP_009785538.1
**77**	54951	55724	257	27.82	Tail assembly protein I	*Pseudoalteromonas* phage KB12-38	6e-09	26.09%	79%	ASV43936.1
**78**	55721	56038	105	12.71	Tail assembly protein	*Pseudoalteromonas* phage KB12-38	1e-13	39.58%	91%	ASV43937.1
**79**	56028	56507	159	18.57	NlpC/P60 family protein	*Achromobacter* phage JWF	5e-13	33.61%	75%	YP_009224029.1
**80**	56523	57002	159	18.27	Minor tail protein L	*Achromobacter* phage JWF	2e-12	29.32%	81%	YP_009224028.1
**81**	56995	57882	295	34.27	Hypothetical protein	*Pseudoalteromonas* phage KB12-38	4e-04	32.03%	41%	ASV43940.1
**82**	57884	64144	2086	226.93	Tail tape measure protein	*Pseudoalteromonas* phage KB12-38	6e-65	31.29%	27%	ASV43941.1
**83**	64137	64340	67	7.7	No similarity	-	-	-	-	-
**84**	64430	64831	133	15.14	Hypothetical protein	*Siphoviridae sp.*	5e-40	53.12%	96%	QHJ78644.1
**85**	64887	65759	290	31.22	Hypothetical protein	*Siphoviridae sp.*	9e-136	70.99%	90%	QHJ78645.1
**86**	65740	66243	167	18.85	Hypothetical protein	*Siphoviridae sp.*	4e-44	47.13%	94%	QHJ78646.1
**87**	66240	67205	321	36.71	Hypothetical protein	*Siphoviridae sp.*	1e-41	32.44%	97%	QHJ78647.1

**88**	67198	67758	186	21.19	Hypothetical protein	*Siphoviridae sp.*	5e-66	54.95%	97%	QHJ78648.1
**89**	67758	68465	235	25.98	Hypothetical protein	*Siphoviridae sp.*	3e-60	43.81%	96%	QHJ78649.1
**90**	68473	68943	156	16.86	Hypothetical protein	*Siphoviridae sp.*	3e-06	33.33%	67%	QHJ78650.1
**91**	69023	70231	402	43.76	Capsid protein	*Pseudoalteromonas* phage KB12-38	3e-44	32.27%	85%	ASV43950.1
**92**	70247	70660	137	14.25	Hypothetical protein	*Siphoviridae sp.*	2e-22	42.11%	97%	QHJ78652.1
**93**	70698	71849	383	41.28	DNA-binding protein	*Pseudoalteromonas* phage KB12-38	1e-30	32.17%	89%	ASV43952.1
**94**	71861	73369	502	55.6	Hypothetical protein	*Siphoviridae sp.*	0	62.15%	100%	QHJ78654.1
**95**	73369	75129	586	67.4	Terminase large subunit	*Achromobacter* phage JWF	1e-127	37.97%	96%	YP_009224013.1
**96**	75460	76212	250	29.05	Hypothetical protein	*Siphoviridae sp.*	*4e-28*	31.40%	96%	QHJ78656.1
**97**	76217	78898	893	102.42	DNA primase	*Caulobacter* phage Seuss	2e-124	30%	97%	YP_009785516.1
**98**	78903	79064	53	6.25	No similarity	-	-	-	-	-
