# Supplementary figures and images for: Isolation and Characterization of a Novel Siphoviridae Phage, vB_AbaS_TCUP2199, Infecting Multidrug-Resistant Acinetobacter baumannii
Source: Viruses. 2022 Jun 7;14(6):1240. doi: 10.3390/v14061240 (PMC9228384; doi:10.3390/v14061240)

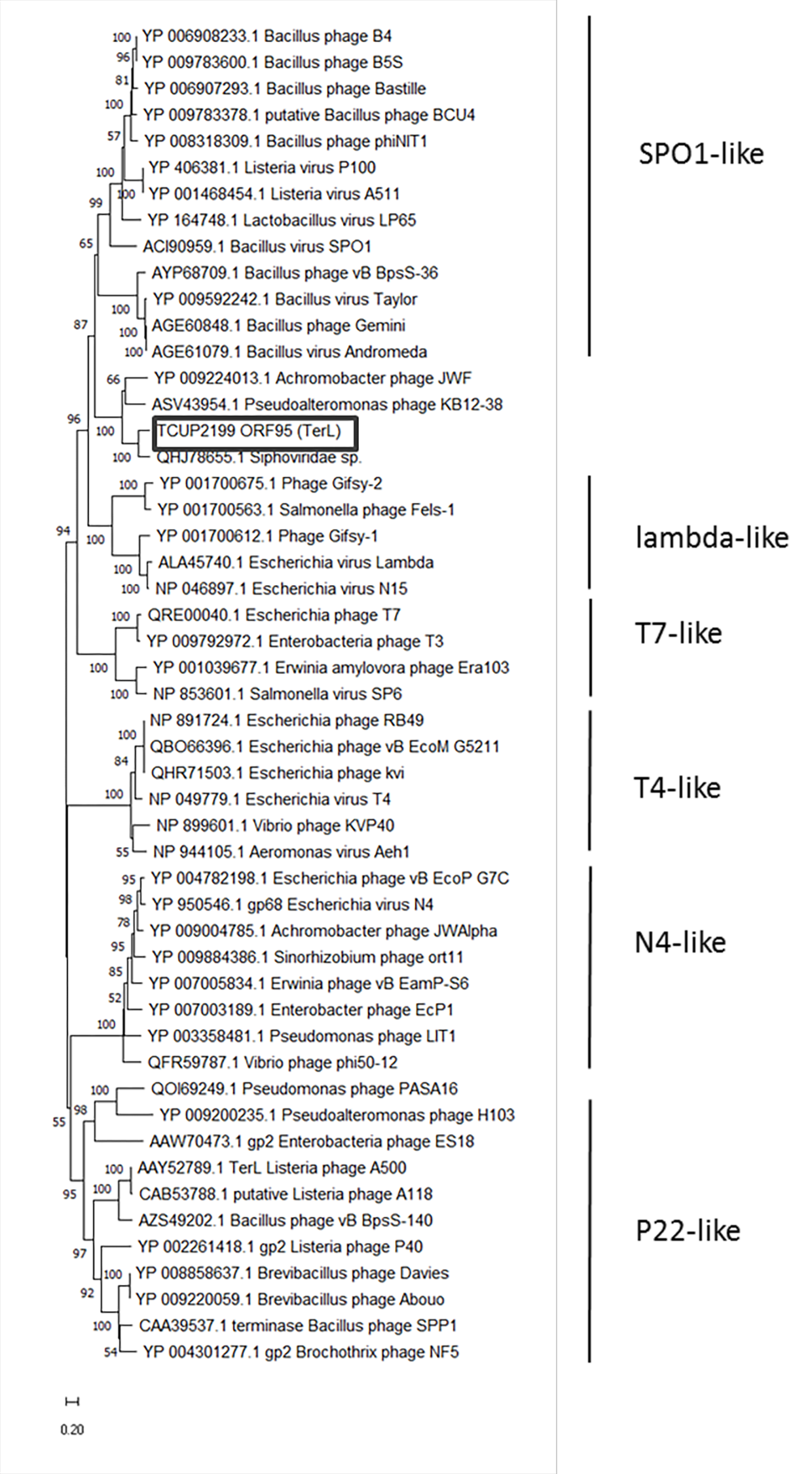

Supplement: Supplementary file 1 [file viruses-14-01240-s001.zip › Figure S1.tif]

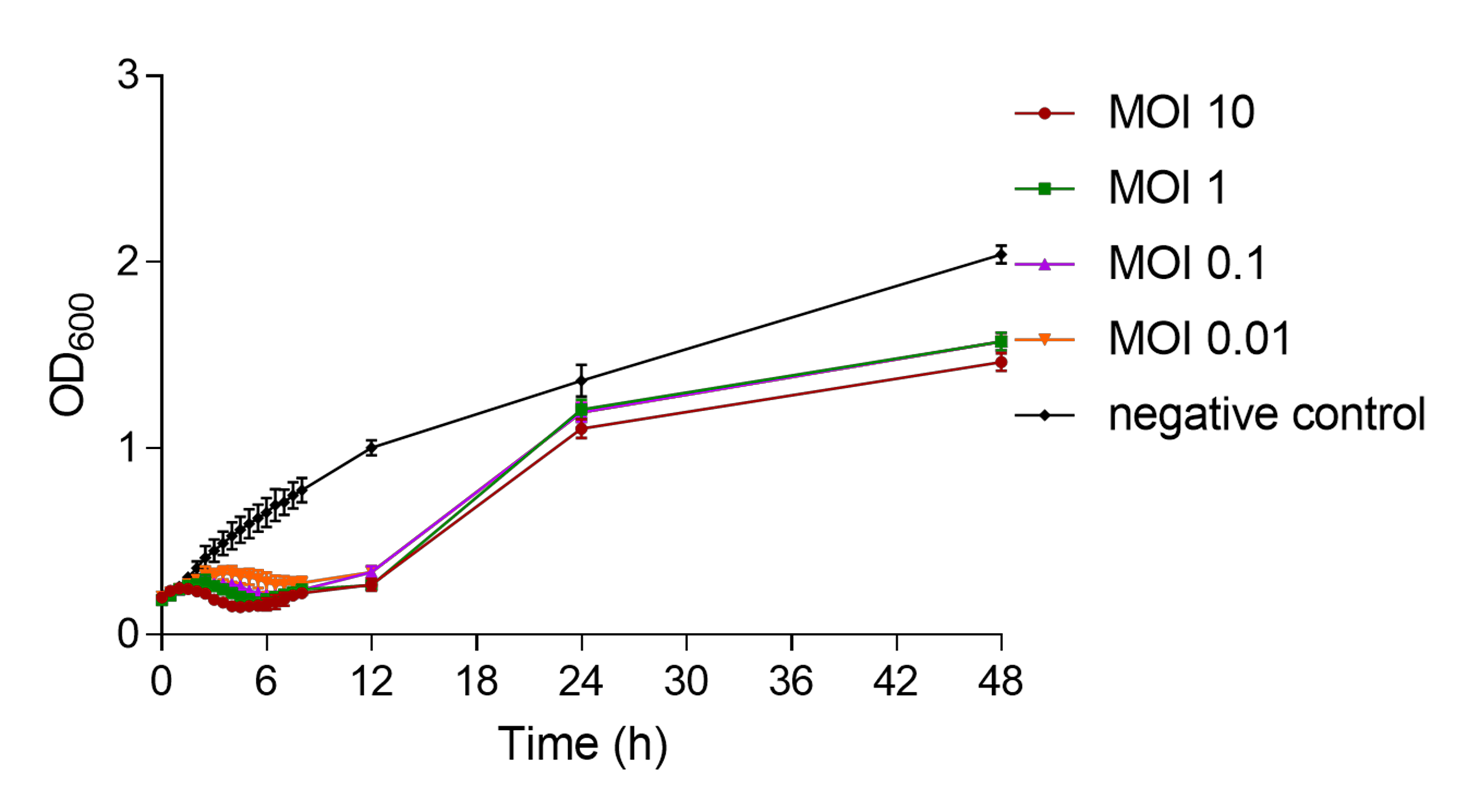

Supplement: Supplementary file 1 [file viruses-14-01240-s001.zip › Figure S2.tif]
